# PGC-1alpha Down-Regulation Affects the Antioxidant Response in Friedreich's Ataxia

**DOI:** 10.1371/journal.pone.0010025

**Published:** 2010-04-07

**Authors:** Daniele Marmolino, Mario Manto, Fabio Acquaviva, Paola Vergara, Ajay Ravella, Antonella Monticelli, Massimo Pandolfo

**Affiliations:** 1 Laboratoire de Neurologie Expérimentale, Université Libre de Bruxelles (ULB), Brussels, Belgium; 2 Fonds National de la Recherche Scientifique (FNRS), Brussels, Belgium; 3 Department of Cellular and Molecular Biology, University of Naples “Federico II”, Naples, Italy; 4 IEOS, Consiglio Nazionale delle Ricerche (CNR), Naples, Italy; Hospital Vall d'Hebron, Spain

## Abstract

**Background:**

Cells from individuals with Friedreich's ataxia (FRDA) show reduced activities of antioxidant enzymes and cannot up-regulate their expression when exposed to oxidative stress. This blunted antioxidant response may play a central role in the pathogenesis. We previously reported that Peroxisome Proliferator Activated Receptor Gamma (PPARγ) Coactivator 1-alpha (PGC-1α), a transcriptional master regulator of mitochondrial biogenesis and antioxidant responses, is down-regulated in most cell types from FRDA patients and animal models.

**Methodology/Principal Findings:**

We used primary fibroblasts from FRDA patients and the knock in-knock out animal model for the disease (KIKO mouse) to determine basal superoxide dismutase 2 (SOD2) levels and the response to oxidative stress induced by the addition of hydrogen peroxide. We measured the same parameters after pharmacological stimulation of PGC-1α. Compared to control cells, PGC-1α and SOD2 levels were decreased in FRDA cells and did not change after addition of hydrogen peroxide. PGC-1α direct silencing with siRNA in control fibroblasts led to a similar loss of SOD2 response to oxidative stress as observed in FRDA fibroblasts. PGC-1α activation with the PPARγ agonist (Pioglitazone) or with a cAMP-dependent protein kinase (AMPK) agonist (AICAR) restored normal SOD2 induction. Treatment of the KIKO mice with Pioglitazone significantly up-regulates SOD2 in cerebellum and spinal cord.

**Conclusions/Significance:**

PGC-1α down-regulation is likely to contribute to the blunted antioxidant response observed in cells from FRDA patients. This response can be restored by AMPK and PPARγ agonists, suggesting a potential therapeutic approach for FRDA.

## Introduction

Friedreich's ataxia (FRDA) is an autosomal recessive inherited disorder affecting approximately 1 in every 40 000 individuals [1] in Western Europe. It is characterized by progressive gait and limb ataxia, dysarthria, areflexia, loss of vibratory and position sense, and progressive weakness of central origin. Additional features include scoliosis, high risk of diabetes [2]–[6] and a hypertrophic cardiomyopathy that can cause premature death [3], [4], [7]. Age of onset is usually in childhood or adolescence, but it may vary from infancy to adulthood.

A large GAA repeat expansion in the first intron of the *FXN* gene is the most common mutation underlying FRDA [8]. Patients are homozygous for this mutation, or, rarely, are compound heterozygotes for the GAA repeat expansion and a different *FXN* loss-of-function mutation. They show severely reduced levels of the *FXN*-encoded mitochondrial protein frataxin, a highly conserved protein with homologs in all eukaryotes and in Gram-negative bacteria [8]. *FXN* down-regulation has been linked to the property of long GAA repeats to adopt a triple helical structure that directly impedes transcription *in vitro* [9], [10]. In the nucleus of cells from human patients and mouse models, *FXN* silencing is associated with epigenetic marks of transcription repressive heterochromatin near expanded GAA repeats [11], [12]. Whether the triplex forming ability of GAA repeats is involved in this chromatin remodeling process is unknown.

Frataxin is an essential protein in higher organisms, as first revealed by the embryonic lethality of *fxn* gene knockout in the mouse [13]. Yeast cells can instead survive without frataxin, but they progressively lose mitochondrial function and mitochondrial DNA [14].

The function of frataxin has not yet been completely elucidated, but its involvement in mitochondrial iron metabolism is supported by current literature. Frataxin has a compact globular structure with functionally important surface features, in particular a negatively charged ridge that binds ferrous iron with low affinity [15], [16]. Under conditions of iron excess, frataxin has been reported to show ferroxidase activity and form high molecular weight complexes containing a ferric iron core [17]. This property, which has been proposed to be important for iron detoxification in the mitochondrial compartment, is most evident for the yeast frataxin homolog yfh1. The functional role of these iron-containing frataxin polymers, as well as of frataxin oligomers reported to form at lower iron concentration, is still controversial [18], [19]. Multiple abnormalities of iron metabolism occur when frataxin levels are insufficient: decreased activities of iron-sulfur cluster (ISC) containing proteins [20], accumulation of iron in mitochondria and depletion in the cytosol [21], enhanced cellular iron uptake [22], [23], and, in some models, reduced heme synthesis [24], [25]. These abnormalities point to a defective utilization of iron for biosynthetic processes taking place in the mitochondria, in particular ISC synthesis. ISCs are prosthetic groups for several mitochondrial and extra-mitochondrial enzymes, involved in energy metabolism (aconitase and complexes I, II and III of the respiratory chain), iron metabolism (iron-responsive protein 1, IRP1, and ferrochelatase), purine metabolism (xanthine oxidase) and DNA repair [26]. Accumulation of iron in the mitochondria with increased cellular uptake and cytosolic depletion occurs when ISC synthesis is defective, suggesting a role of frataxin in this process. Current evidence supports a direct interaction of frataxin with components of the mitochondrial ISC synthesis machinery, but a non-essential role in the process [27].

Evidence of oxidative stress has been found in most, though not all models of frataxin deficiency [28]–[35]. In FRDA patients, increased plasma levels of malonyldialdehyde (a lipid peroxidation product) [34], increased urinary 8-hydroxy-2'-deoxyguanosine (a marker of oxidative DNA damage) [35], decreased plasma free glutathione, and increased plasma glutathione *S*-transferase activity [29] indicate an oxidative stress condition. Oxidative stress is thought to derive from the strong reactivity of the excess mitochondrial iron with reactive oxygen species (ROS) present in that compartment, including the Fenton reaction that generates the highly toxic hydroxyl radical. Accordingly, yfh1-deficient yeast [14] and cells from FRDA patients [36] are highly sensitive to oxidants such as hydrogen peroxide H_2_O_2_. Respiratory chain dysfunction caused by decreased activity of the ISC-containing complexes I, II and III is likely to further aggravate oxidative stress by increasing leakage of electrons and formation of superoxide.

Frataxin deficient cells not only generate more free radicals, they also show a reduced ability to mobilize antioxidant defenses, in particular to induce SOD2 expression following exposure to oxidants such as H_2_O_2_ and iron [31]. The mechanism underlying this defect has not yet been understood. Its investigation has prompted the present study.

In a previous study we have shown reduced expression of the peroxisome proliferator activated receptor gamma (PPAR-γ) coactivator 1α (PGC-1α) in several tissues from frataxin-deficient mice, with the notable exception of the heart. We have observed PGC-1α down-regulation also in neural precursor cells from the subventricular zone of these animals, and in fibroblasts and lymphoblastoid cell lines from FRDA patients [37], [38]. PGC-1α is a multifunctional protein found at higher levels in tissues with high metabolic requirement such as brown fat, skeletal muscle, kidney, heart, and brain [39]–[41], that functions as a coactivator to most nuclear receptors and to several other transcription factors [42]. It is a critical regulator that links metabolic activity to relevant environmental stimuli in multiple pathways, including those responsible for adipogenesis, gluconeogenesis, myogenesis, and mitogenesis [43]. PGC-1α has also emerged as a key factor in the induction of many antioxidant programs in response to oxidative stress, both *in vivo* and *in vitro* [44]–[47], in particular in neurons [46]. RNAi knockdown of *Pgc1a* prevents the induction by ROS of antioxidant enzymes such as superoxide dismutase 1 (*Sod1*), superoxide dismutase 2 (*Sod2*), and *Gpx1*, as well as the uncoupling proteins *Ucp1* and *Ucp2* [46], indicating that it mediates these protective responses [46], [47].

We further observed that in C2C12 myoblasts, but not in cardiomyocytes, PGC-1α and a reporter gene under the control of the PGC-1α promoter are rapidly down-regulated when frataxin expression is inhibited by an shRNA [37], indicating that some mechanism directly links an early effect of frataxin deficiency with reduced PGC-1α transcription in this cell type, and presumably in other cells that also down-regulate PGC-1α when frataxin levels are low.

In this study we tested whether the PGC-1α down-regulation occurring in FRDA cells could be in part responsible for the blunted antioxidant response observed in frataxin-deficiency. Our results support this hypothesis, indicating a possible therapeutic target in FRDA.

## Results

### PGC-1α reduction is associated to reduced SOD2 in FRDA fibroblasts and does not increase after H_2_O_2_ incubation

**Figure 1:**
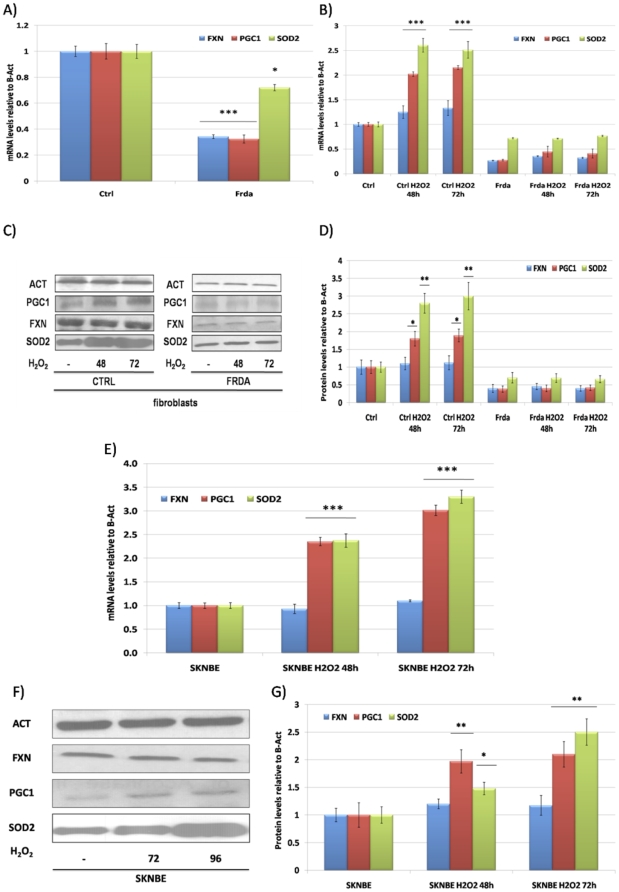
SOD2 and PGC-1α expression in FRDA fibroblasts and H_2_O_2_ treatments. **A.** Quantitative Real-Time PCR analysis: FXN (Blue bars), PGC-1α (Red bars) and SOD2 (Green bars) mRNA quantification in primary fibroblasts from healthy controls and FRDA patients. **B.** Quantitative Real-Time PCR analysis: FXN (Blue bars), PGC-1α (Red bars) and SOD2 (Green bars) mRNA quantification in primary fibroblasts from healthy controls and FRDA patients after incubation with 100 µM H_2_O_2_ at 48 and 72 hours. **C.** Western Blot analysis: β-Actin (Act), PGC-1α (PGC-1), frataxin (FXN), mitochondrial superoxide dismutase (SOD2) protein in primary fibroblasts from healthy controls and FRDA patients after incubation with 100 µM H_2_O_2_ at 48 and 72 hours. **D.** Densitometric scan analysis of five independent Western blots from healthy controls and FRDA patients after incubation with 100 µM H_2_O_2_ at 48 and 72 hours: FXN (Blue bars), PGC-1α (Red bars) and SOD2 (Green bars). The relative intensities of the bands were quantified using the Image J Software, and all the values were normalized to the intensities of the respective β-Actin signal. **E.** Quantitative Real-Time PCR analysis: FXN (Blue bars), PGC-1α (Red bars) and SOD2 (Green bars) mRNA quantification in SKNBE neuroblastoma cells after incubation with 100 µM H_2_O_2_ at 48 and 72 hours. **F.** Western Blot analysis: β-Actin (Act), PGC-1α (PGC-1), frataxin (FXN), mitochondrial superoxide dismutase (SOD2) protein in SKNBE neuroblastoma cells after incubation with 100 µM H_2_O_2_ at 48 and 72 hours. **G.** Densitometric scan analysis of five independent Western blots from SKNBE cells after incubation with 100 µM H_2_O_2_ at 48 and 72 hours: FXN (Blue bars), PGC-1α (Red bars) and SOD2 (Green bars). The relative intensities of the bands were quantified using the Image J Software, and all the values were normalized to the intensities of the respective β-Actin signal. Results are expressed as a fold increase of the means (mean ± SEM) over the value of expression in the respective untreated control cells arbitrarily set as 1. (n = 5, ***p<0.001, **p<0.01,*p<0.05; Mean +/− SEM) for all the experiments.

The baseline expression levels of SOD2 and PGC-1α were reduced in fibroblasts from FRDA patients when compared to healthy controls cells (Fig. 1A). Treatment with 100 µM H_2_O_2_ for 48 and 72 hours increased both SOD2 and PGC-1α mRNA and protein in control cells, but not in FRDA fibroblasts (Fig. 1B–D). Incubation with H_2_O_2_ of SKNBE neuroblastoma cells was also accompanied by PGC-1α and SOD2 induction at both mRNA and protein level, showing that this response is not specific for fibroblasts (Fig. 1E–G).

### PGC-1α down-regulation by RNAi results in lack of SOD2 response to H_2_O_2_

**Figure 2:**
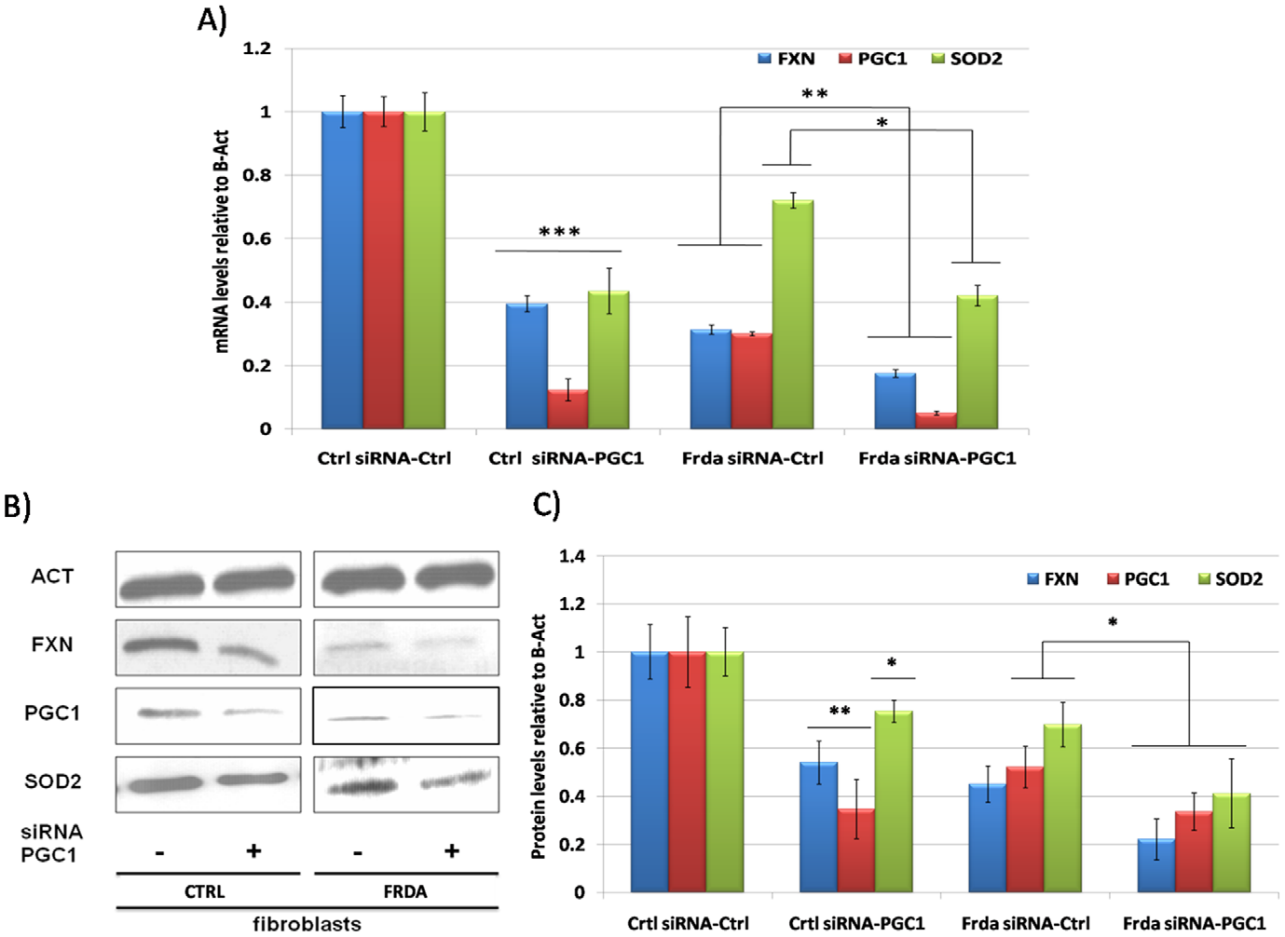
PGC-1α downregulation by a specific RNAi. **A.** Quantitative Real-Time PCR analysis: FXN (Blue bars), PGC-1α (Red bars) and SOD2 (Green bars) mRNA quantification in primary fibroblasts from healthy controls and FRDA patients after 72 hours transfection with a PGC-1α specific siRNA, as control a fluoresceine-conjugated siRNA was used. **B.** Western Blot analysis: β-Actin (Act), PGC-1α (PGC-1), frataxin (FXN), mitochondrial superoxide dismutase (SOD2) protein in primary fibroblasts from healthy controls and FRDA patients after 72 hours transfection with a PGC-1α specific siRNA, as control a fluoresceine-conjugated siRNA was used. **C.** Densitometric scan analysis of five independent Western blots from healthy controls and FRDA patients after 72 hours transfection with a PGC-1α specific siRNA, as control a fluoresceine-conjugated siRNA was used: FXN (Blue bars), PGC-1α (Red bars) and SOD2 (Green bars). The relative intensities of the bands were quantified using the Image J Software, and all the values were normalized to the intensities of the respective β-Actin signal. (n = 5, ***p<0.001, **p<0.01, *p<0.05; Mean +/− SEM) for all the experiments.

Incubation of fibroblasts from healthy and FRDA subjects for 72 hours with a PGC-1α specific siRNA significantly decreases mRNA and protein levels when compared to cells transfected with a control non-specific siRNA, as shown in figure 2A–C. In these conditions, SOD2 mRNA and protein reduction was significant both in control and FRDA fibroblasts. These data are in agreement with reports indicating that PGC-1α controls SOD2 expression [47], [48], [49]. We also confirmed our previous finding [37] that PGC-1α silencing results in frataxin down-regulation.

### Effect of PPARγ and AMPK agonists on the antioxidant response in FRDA fibroblasts

We tested whether drugs known to up-regulate PGC-1α could restore SOD2 induction by H_2_O_2_ in FRDA cells. PPARγ and the AMP-dependent protein kinase (AMPK) are major inducers of PGC-1α activity and expression [50]–[54].

**Figure 3:**
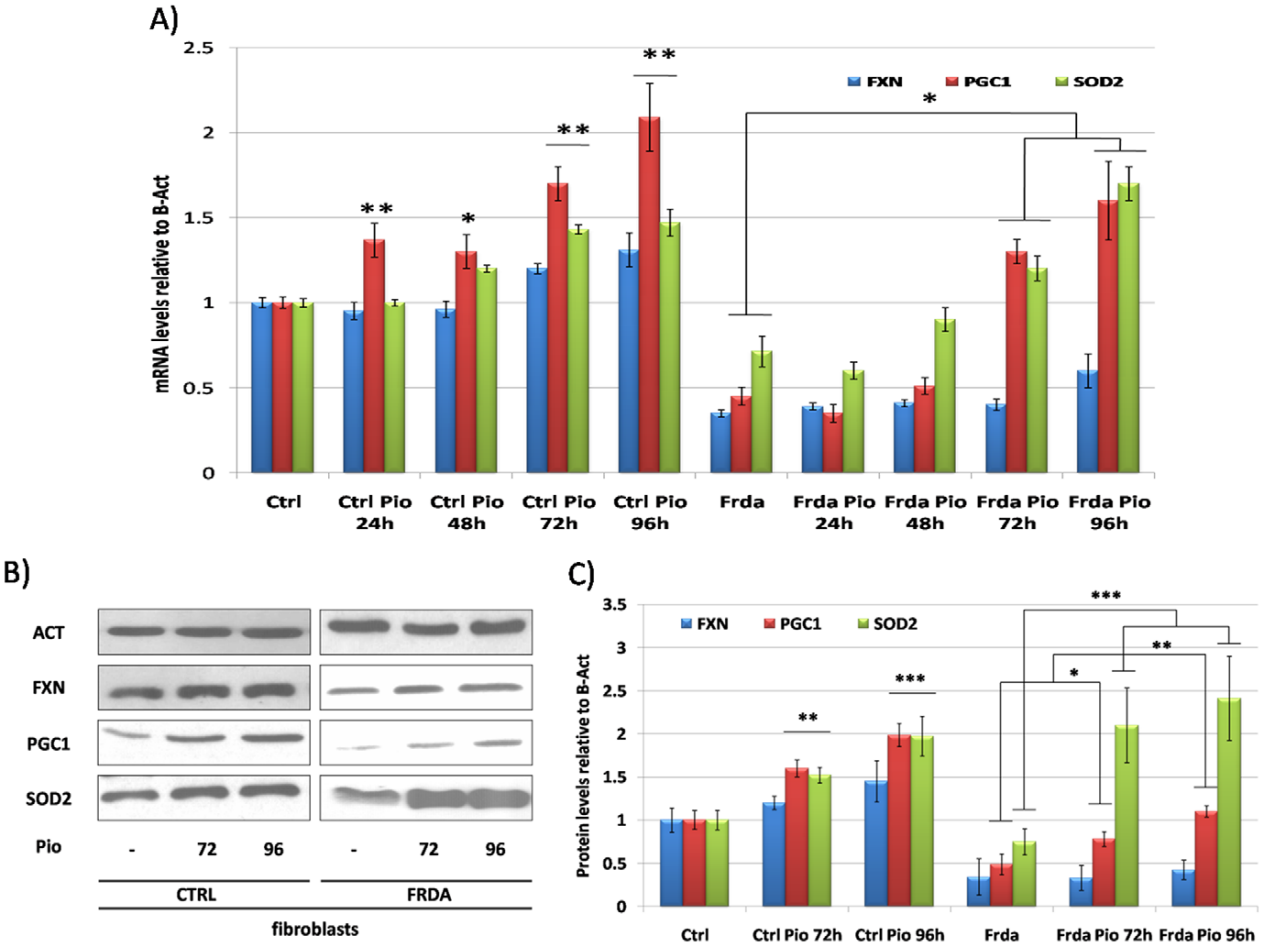
Effect of Pioglitazone on the antioxidant response in FRDA fibroblasts and SKNBE neuroblastoma cells. **A.** Quantitative Real-Time PCR analysis: FXN (Blue bars), PGC-1α (Red bars) and SOD2 (Green bars) mRNA quantification in primary fibroblasts from healthy controls and FRDA patients after incubation with 10 µM Pioglitazone at 24, 48, 72 and 96 hours. **B.** Western Blot analysis: β-Actin (Act), PGC-1α (PGC-1), frataxin (FXN), mitochondrial superoxide dismutase (SOD2) protein in primary fibroblasts from healthy controls and FRDA patients after incubation with 10 µM Pioglitazone at 72 and 96 hours. **C.** Densitometric scan analysis of five independent Western blots from healthy controls and FRDA patients after incubation with 10 µM Pioglitazone at 72 and 96 hours: FXN (Blue bars), PGC-1α (Red bars) and SOD2 (Green bars). The relative intensities of the bands were quantified using the Image J Software, and all the values were normalized to the intensities of the respective β-Actin signal. **D.** Quantitative Real-Time PCR analysis: FXN (Blue bars), PGC-1α (Red bars) and SOD2 (Green bars) mRNA quantification in SKNBE neuroblastoma cells after incubation with 10 µM Pioglitazone at 72 and 96 hours. **E.** Western Blot analysis: β-Actin (Act), PGC-1α (PGC-1), frataxin (FXN), mitochondrial superoxide dismutase (SOD2) protein in SKNBE neuroblastoma cells after incubation with 10 µM Pioglitazone at 72 and 96 hours. **F.** Densitometric scan analysis of five independent Western blots from SKNBE cells after incubation with 10 µM Pioglitazone at 72 and 96 hours: FXN (Blue bars), PGC-1α (Red bars) and SOD2 (Green bars). The relative intensities of the bands were quantified using the Image J Software, and all the values were normalized to the intensities of the respective β-Actin signal. (n = 5, ***p<0.001, **p<0.01,*p<0.05; Mean +/− SEM) for all the experiments.

In a first set of experiments we evaluated the effect of the PPARγ agonist Pioglitazone. We had previously shown that a different potent PPARγ agonist, Azelaoyl-PAF, was able to increase PGC-1α and frataxin expression in FRDA and control fibroblasts [55]. Pioglitazone at a concentration of 10 µM was able to increase PGC-1α mRNA and protein levels after 72 and 96 hours of incubation of both control and FRDA fibroblasts, as shown in Fig. 3A–C. In the same conditions, SOD2 levels were also increased, with a more robust effect in FRDA cells. Frataxin expression showed a non-significant trend toward up-regulation in this set of experiments (Fig. 3A–C). Interestingly, in SKNBE neuroblastoma cells, Pioglitazone incubation for 96 hours at 10 µM increased PGC-1α and SOD2 levels as well as frataxin amount (Fig. 3D–F).

**Figure 4:**
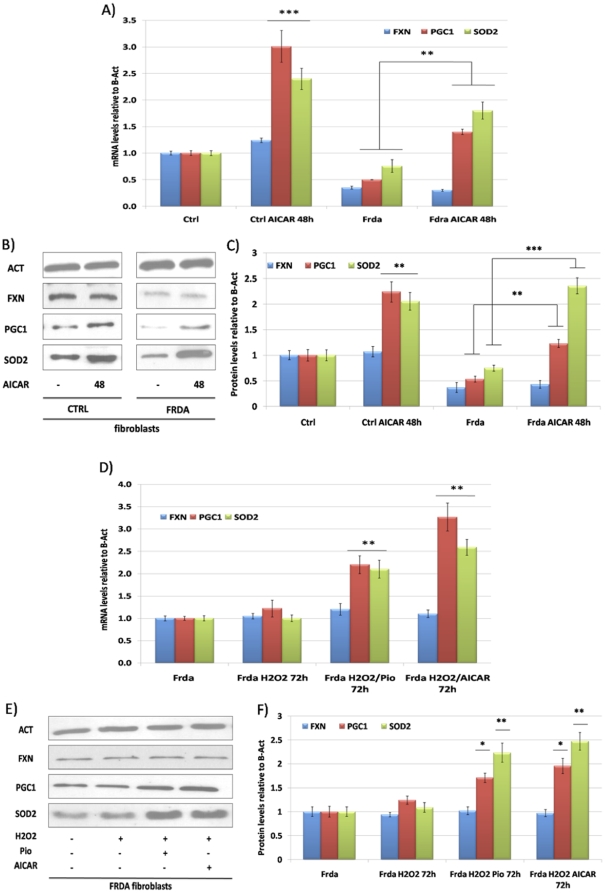
Effect of AICAR on the antioxidant response in FRDA fibroblasts and SKNBE neuroblastoma cells. **A.** Quantitative Real-Time PCR analysis: FXN (Blue bars), PGC-1α (Red bars) and SOD2 (Green bars) mRNA quantification in primary fibroblasts from healthy controls and FRDA patients after incubation with 2 mM AICAR at 48 hours. **B.** Western Blot analysis: β-Actin (Act), PGC-1α (PGC-1), frataxin (FXN), mitochondrial superoxide dismutase (SOD2) protein in primary fibroblasts from healthy controls and FRDA patients after incubation with 2 mM AICAR at 48 hours. **C.** Densitometric scan analysis of five independent Western blots from healthy controls and FRDA patients after incubation with 2 mM AICAR at 48 hours: FXN (Blue bars), PGC-1α (Red bars) and SOD2 (Green bars). The relative intensities of the bands were quantified using the Image J Software, and all the values were normalized to the intensities of the respective β-Actin signal. **D.** Quantitative Real-Time PCR analysis: FXN (Blue bars), PGC-1α (Red bars) and SOD2 (Green bars) mRNA quantification in primary fibroblasts from FRDA patients after incubation with 100 µM H_2_O_2_ alone or in combination with 10 µM Pioglitazone or 2 mM AICAR for 72 hours. **E.** Western Blot analysis: β-Actin (Act), PGC-1α (PGC-1), frataxin (FXN), mitochondrial superoxide dismutase (SOD2) protein in primary fibroblasts from FRDA patients after incubation with 100 µM H_2_O_2_ alone or in combination with 10 µM Pioglitazone or 2 mM AICAR for 72 hours. **F.** Densitometric scan analysis of five independent Western blots from SKNBE cells after incubation with 100 µM H_2_O_2_ alone or in combination with 10 µM Pioglitazone or 2 mM AICAR for 72 hours: FXN (Blue bars), PGC-1α (Red bars) and SOD2 (Green bars). The relative intensities of the bands were quantified using the Image J Software, and all the values were normalized to the intensities of the respective β-Actin signal. (n = 5, ***p<0.001, **p<0.01, *p<0.05; Mean +/− SEM) for all the experiments.

We then exposed cells to the AMPK agonist AICAR, showing that this molecule at a concentration of 2 mM, strongly up-regulates PGC-1α and SOD2 in both healthy controls and FRDA fibroblasts after 48 hours of incubation (Fig. 4A–C).

Accordingly, Pioglitazone 10 µM or AICAR 2 mM, incubation for 5 hours with a following addiction of 100 µM H_2_O_2_ for 72 hours increase SOD2 expression in FRDA fibroblasts (Fig. 4D–F). In these conditions, an increase in PGC-1α levels was also observed when compared to treatment with H_2_O_2_ alone (Fig. 4D–F).

### Effect of Pioglitazone *in vivo* in frataxin-deficient (KIKO) mice

**Figure 5:**
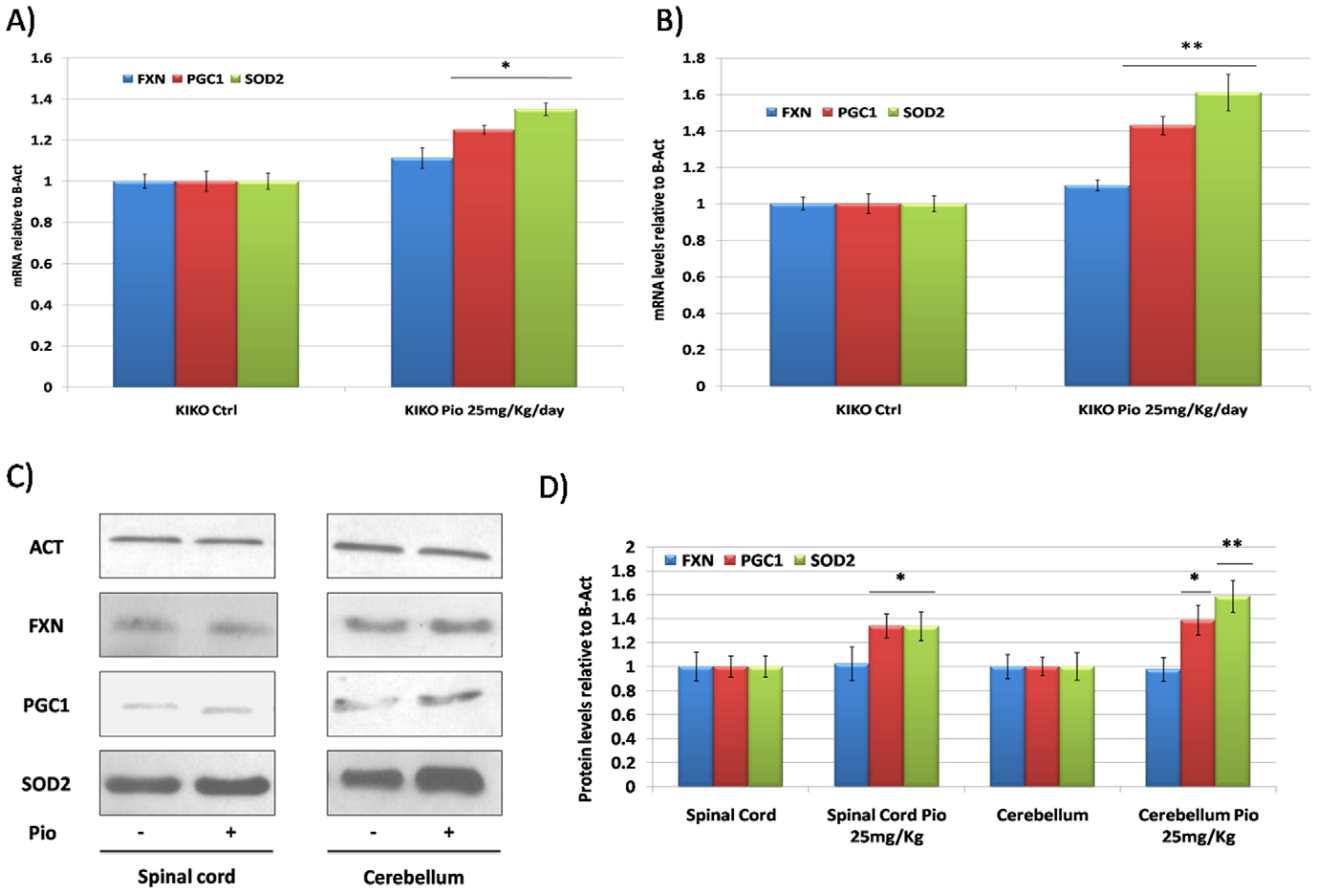
*In vivo* Pioglitazone administration in KIKO mice. **A.** Quantitative Real-Time PCR analysis: *fxn* (Blue bars), *pgc-1α* (Red bars) and *sod2* (Green bars) mRNA quantification in the spinal cord of the KIKO mice after receiving oral administration of Pioglitazone 25 mg/Kg/day for one month. **B.** Quantitative Real-Time PCR analysis: *fxn* (Blue bars), *pgc-1α* (Red bars) and *sod2* (Green bars) mRNA quantification in the cerebellum of the KIKO mice after receiving oral administration of Pioglitazone 25 mg/Kg/day for one month. **C.** Western Blot analysis: *β-actin* (Act), *pgc-1α* (PGC-1), *frataxin* (FXN), mitochondrial superoxide dismutase (*sod2*) protein in the spinal cord and cerebellum of the KIKO mice after receiving oral administration of Pioglitazone 25 mg/Kg/day for one month. **D.** Densitometric scan analysis of five independent Western blots from the spinal cord and cerebellum of the KIKO mice after receiving oral administration of Pioglitazone 25 mg/Kg/day for one month: FXN (Blue bars), PGC-1α (Red bars) and SOD2 (Green bars). The relative intensities of the bands were quantified using the Image J Software, and all the values were normalized to the intensities of the respective β-Actin signal. (n = 10, **p<0.01,*p<0.05; Mean +/− SEM) for all the experiments.

Based on *in vitro* results, we tested the effect of Pioglitazone on the levels of PGC-1α, SOD2 and frataxin in frataxin-deficient mice. For these experiments, we used the fxn^(GAA)230/−^ (KIKO) mice that express 25–35% of wild-type frataxin levels, but have no detectable motor abnormality of pathological change. Ten KIKO mice received Pioglitazone (25 mg/Kg/day) via oral administration for one month. No change was observed in the body weight of the mice (data not shown). In the spinal cord, a primary affected tissue in the disease, no effect was observed on frataxin expression, while Pgc-1α and Sod2 levels were slightly increased (Fig. 5A–D). In the cerebellum, Pgc-1α and Sod2 levels were significantly increased (Fig. 5B–D). No effect was observed on frataxin expression. Surprisingly, in a group of 10 wt mice no effect was observed after Pioglitazone administration.

## Discussion

Our results provide a link between the previous independent observations of a blunted antioxidant response in cells from FRDA patients [31], [32] and the PGC-1α down-regulation occurring in most cell types with frataxin deficiency [37]. These phenomena are likely to contribute to FRDA pathogenesis and constitute possible therapeutic targets.

Oxidative stress has been considered a major pathogenic mechanism in FRDA, even though the data are in same case controversial. Previous studies [56], [57] found no evidence of oxidative stress in the target tissues of conditional knockout mouse models (heart and nervous system), which nevertheless develop FRDA-like pathology and show the typical biochemical defects of FRDA, including multiple ISC enzyme deficiencies and, in late stages, gross mitochondrial iron accumulation. One possible explanation could be that the total absence of frataxin, as found in targeted cells in conditional knock-outs, leads to an almost complete respiratory chain shut down, so less ROS are eventually generated. Indeed, in yeasts studies evidence of oxidative damage rapidly follows frataxin silencing before the loss of mitochondrial function [58]. The respiratory chain, though impaired, remains partially functional in FRDA target tissues and in animal models with reduced frataxin levels, as well as in cell models with partial frataxin deficiency or expressing a mutated frataxin protein [59], [60]. Accordingly, studies in these systems [59], [61], [62], including tissue samples from FRDA patients, have shown evidence of chronic oxidative stress, and oxidative stress markers have been found in the blood [29], [34] and urine [35] of FRDA patients. In the case of the fibroblasts utilized in the present study, we obtained further evidence of increased ROS production by revealing higher levels of superoxide than in control fibroblasts (Methods S1 and Fig. S1). Then, we confirmed [31], [32] that exposure of FRDA fibroblasts to moderate oxidative stress, as induced by exogenously added H_2_O_2_ or iron, or by partial respiratory chain inhibition, fails to up-regulate antioxidant enzymes. Only strong stressors, like very high iron or H_2_O_2_ concentration in the medium have shown to possibly up-regulate SOD2 in FRDA fibroblasts [31] by triggering NFκB signaling. These data indicate the failure of a response mechanisms dealing with the control of lower, chronic levels of oxidative stress. With reference to pathogenesis, it is conceivable that, while this deficient response does not appear to be harmful to unaffected cells in FRDA like fibroblasts, unless they are exposed to additional oxidative stress, it may be deleterious for vulnerable cell types, such as neurons even in basal conditions.

The observed failure to induce antioxidant defenses contrasts with the expected homeostatic response. Several pathways are physiologically activated by ROS, leading to increased levels and activity of antioxidant enzymes and to mitochondrial biogenesis [42]–[44], [46], [47], [63], [64]. Key factors for antioxidant enzyme induction are nuclear factor-E2-related factor-2 (Nrf2), a transcription factor that serves as a cellular sensor for oxidative stress [64], [65], and PGC-1α [66].

PGC-1α is also a key player in the ROS-induced mitochondrial biogenesis, along with the transcription factor nuclear respiratory factor-1 (NRF-1) and the mitochondrial transcription factor Tfam [48], [66]–[71].

A recent study suggested that impaired nuclear translocation of Nrf2 may underlay the lack of oxidative stress response in FRDA cells [71]. Though this mechanism may play a role, Nrf2-regulated genes primarily include heme oxygenase-1 (HO-1), NAD(P)H:quinone oxidoreductase-1 (NQO1), glutathione S-transferases, and the glutathione-synthesizing enzymes glutamate-cysteine ligase catalytic subunit (GCLC) and glutamate-cysteine ligase modifier subunit (GCLM), while PGC-1α may be more relevant for SOD2 induction.

The importance of PGC-1α in these metabolic programs was further revealed through the generation of PGC-1a null mice. These mice display a reduced basal expression of many mitochondrial genes in liver, brain, skeletal muscle, and heart compared with wild-type (WT) animals [72]–[74]. Furthermore, PGC-1α knockout (KO) mice underlying PGC-1a as an important factor in brain structure and function. PGC-1α KO animals present neurodegenerative lesions in the striatum, as well as behavioral abnormalities [74]. These lesions present characteristic similar to those observed in many models with altered ROS levels. Thus, PGC-1α could play an important role in ROS control. The precise role of PGC-1a in ROS metabolism is still undiscovered. Several groups have reported that the expression of mitochondrial ROS-detoxifying enzymes increases with PGC-1α [75]–[77]. Conversely, muscle from PGC-1α knockout mice shows a mild reduction of SOD2 [78]. Interestingly, PGC-1α direct down-regulation by RNAi results in the downregulation of SOD2 and other antioxidant enzymes, and particularly generates a lack of their induction after exposure to stressors such as H_2_O_2_, markedly resembling the situation in frataxin-deficient cells [46]. This important finding, together with our previous observation that frataxin deficiency leads to reduced levels of PGC-1α and its target genes in most investigated cell types prompted us to study whether PGC-1α could be involved in the blunted antioxidant response in FRDA.

We show that lack of PGC-1α induction parallels the lack of SOD2 induction in FRDA fibroblasts exposed to H_2_O_2_, that PGC-1α silencing by siRNA in normal fibroblasts mimics the lack of antioxidant response found in FRDA cells, and that pharmacological PGC-1α up-regulation obtained by stimulating its activators PPARγ or AMPK can restore the SOD2 response in H_2_O_2_ stressed FRDA cells. In addition, we show that *in vivo* treatment of KIKO mice with the PPARγ agonist Pioglitazone, known to cross the blood-brain barrier, increased SOD2 levels both in the spinal cords and cerebellum. Those tissues are primary affected in patients. Taken together, these results raise the hypothesis that the PGC-1α repression observed in FRDA cells could underlie the lack of antioxidant response. We also show that two pathways that can induce PGC-1α remain functional and can be stimulated in order to stimulate a down-stream response.

Similar results were obtained in a study on an animal model of a mitochondrial disorder, a skeletal muscle conditional knock-out mouse for the gene encoding the cytochrome c oxidase assembly factor COX10. In that model as well, mitochondrial biogenesis and antioxidant responses were not effectively induced unless the PPARγ/PGC-1α pathway was genetically or pharmacologically stimulated [78].

In our experiments, AMPK kinase stimulation resulted in a faster up-regulation of PGC-1α and SOD2 than the PPARγ agonist, but in both cases their levels became close to those in control cells. A possible explanation is that in FRDA cells, in addition to be transcriptionally down-regulated, PGC-1α is also mostly present in a less functional form, possibly due to acetylation and lack of AMPK phosphorylation. Activation of the AMPK pathway would then result in the direct activation of the existing PGC-1α pool by phosphorylation and SIRT1-mediated deacetylation [79], [80], leading to rapidly increased transcription of target genes, including PGC-1α itself. PPARγ stimulation would have instead to first recruit the smaller active PGC-1α pool to co-stimulate transcription of a first set of target genes, again including PGC-1α itself, whose progressively higher abundance would later amplify the response. Together with our previous findings that after frataxin silencing with an shRNA in cultured myoblasts, PGC-1α is rapidly repressed and a reporter gene driven by a PGC-1α promoter is also rapidly down-regulated [37], these data suggest that frataxin deficiency directly leads to PGC-1α inactivation, followed by decreased transcription. AMPK, which is a target of ROS and the main activator of PGC-1α, appears an appealing candidate as the responsible for these events, but direct evidence is still lacking.

## Materials and Methods

### Ethics statement

Patients and healthy controls were enrolled on a voluntary basis at the “Federico II” University in Naples, Italy. Written informed consent to participate in the study and provide a skin biopsy was obtained according to a protocol approved by the “Federico II” ethics committee.

### Patients

The study included five FRDA patients from the department of Neurology “Federico II” Naples, and five unrelated healthy controls. Patients were homozygous for GAA repeat expansions between 500 and 1,200 repeats, with an age of onset for the disease ranging between 20 and 30 years old. All enrolled patients started treatment with Idebenone 5 mg/kg after the skin biopsies were obtained. They were also following a standard protocol of physiotherapy.

### Cell cultures

Fibroblast primary cell cultures were obtained from skin biopsies of FRDA patients and healthy controls. Human neuroblastoma derived cells (SKNBE) are commercially available (in Europe from ATCC-LGC Standards, line number CRL-2271). Primary fibroblasts and SKNBE cells were grown in DMEM supplemented with 15% fetal bovine serum (FBS), 2 mM L-glutamine, 100 U/ml penicillin and streptomycin (P/S). All experiments with fibroblasts were conducted between fourth and eleventh passages.

### Cell Treatment

Primary fibroblasts and SKNBE cells were incubated for 48 and 72 hours in presence of 100 µM H_2_O_2_ (Sigma Aldrich) or DMEM alone as control before total RNA and protein extraction. 10 µM Pioglitazone (AD-4833, Takeda pharmaceuticals) and 2 mM 5-Aminoimidazole-4-carboxamide 1-β-D-ribofuranoside (AICAR, Sigma Aldrich) were used for *in vitro* experiments.

### Animal experiments

20 (C57BL6/j; *fxn*GAA^230/−^) KIKO mice eight months old were used for *in vivo* experiments. Precisely, 10 KIKO mice received 25 mg/Kg/day of Pioglitazone/0.1% carboxymethylcellulose sodium salt (Sigma Aldrich) via oral administration (gavages) for 1 month. A control group of 10 KIKO mice received 0.1% carboxymethylcellulose sodium salt. Mice body weight was daily monitored. Mice were then sacrificed via a cervical dislocation and tissues were extracted for the analysis. All animal procedures respected regulations and guidelines of the Belgian state and European Union and were approved by the animal ethics and welfare committee of the Université Libre de Bruxelles (CEBEA), where the animal experiments were carried out.

### Cells transfections

Primary fibroblasts from both healthy controls and FRDA patients were plated at a concentration of 3×10^5^ well in antibiotic-free DMEM 24 hours before the experiment. One hour before the transfection, cells were growth in OPTI-MEM® (Gibco). For each transfection a mix containing 1 mg of either the *Ppargc1a* ((h) sc-38884; Santa Cruz Biotechnology) or control siRNA fluoresceine-conjugated (sc-37007; Santa Cruz Biotechnology) and Lipofectamine RNAiMAX (Invitrogen) in OPTI-MEM® was prepared. Cells were incubated with the mix for 24 hours at 37°C in presence of CO_2_. Than complete DMEM was added and cells were growth in normal conditions for 72 hours before total RNA and protein extractions. The efficient of the transfection was monitored by fluorescence microscopy for the fluoresceine-conjugated siRNA and RT-PCR for the *Ppargc1* siRNA.

### Real-time quantitative PCR

Total mRNA from primary fibroblasts and SKNBE cells was extracted using the RNeasy mini kit (Qiagen) according to the manufacture's protocol. Mice's tissues were homogenized and total RNA was extracted using the Qiazol reagent and the RNeasy lipid tissues kit (Qiagen) according to the protocol. After a DNAse treatment (RNAse-Free DNAse set, Qiagen), one microgram of total mRNA from cells and tissues was reverse-transcribed with the QuantiTect Reverse Transcription Kit (Qiagen). Approximately 100 ng of cDNA were amplified by Real-Time PCR using the Power SYBR® Green Master mix (Applied Biosystems, Foster City, CA). All the samples were run in triplicate using a 7500 Real Time PCR system (Applied Biosystems, Foster City, CA). Post assay analysis was performed using the SDS software, version 2.3 (Applied Biosystems, CA). qPCR primers are listed below: Human (PPARGC1A: Qiagen (QT00095578); FXN: forward 5′-CAGAGGAAACGCTGGACTCT-3′, reverse 5′-AGCCAGATTTGCTTGTTTGG-3′; MnSOD: Qiagen (QT01008693); Act-b: forward 5′-AGAAAATCTTGGCACCACACC-3′, reverse 5′-AACGGCAGAAGAGAGAACCA-3′; GAPDH: forward 5′- TGCACCACCAACTGCTTAGC -3′, reverse 5′- GGCATGGACTGTGGTCATGAG -3′); Mouse (Ppargc1a: Qiagen (QT00156303); fxn: forward: 5′-CCTGGCCGAGTTCTTTGAA-3′, reverse: 5′-GCCAGATTTGCTTGTTTGG-3′; sod2: Qiagen (QT00161707); act-b: forward: AACCGTGAAAAGATGACCCAGAT; reverse: 5′-GCCTGGATGGCTACGTACATG-3′; rer1: forward: 5′-CCACCTAAACCTTTTCATTGCG-3′, reverse: 5′-TTTGTAGCTGCGTGCCAAAAT-3′). All the results for both human and animal experiments have been normalized using β-actin as reference gene. GAPDH for human samples and rer1 for mouse samples have been additionally tested, in several cases, to confirm the results. Quantitative Real-Time PCR analyses were carried out using the 2(-Delta Delta C(T)) method (2-ΔΔCt).

### Western blot

For total proteins extraction, fibroblasts and SKNBE cells were lysed in a buffer containing 10 mM Tris (pH 7.4), 150 mM NaCl, 1 mM EDTA (pH 8.0), 1% Triton X-100, 1 mM phenylmethylsulfonyl fluoride and 10 µg/ml of leupeptin/aprotinin for 30 minutes on ice. The lysate was sonicated and the concentration was determined using a Bradford assay. 100 mg of total protein were used for the analysis. The running conditions included a 10 min incubation at 10 mA (Volt and Watt at maximum) and then a step at 25 mA for 60–90 min. For blotting, a nitrocellulose membrane was used. Blotting was conducted at 10 W and 250 V (250 mA per gel) for 60 min. After blocking in 5% milk, the membrane was incubated with the primary antibody (Frataxin, 1∶5000; Chemicon; pgc1α, 1∶1000; Cell Signaling; SOD2, 1∶1000 and β-actin, 1∶10000; Sigma Aldrich) lasted overnight at 4°C. After the incubation, the blot was washed and a peroxidase conjugated secondary antibody was incubated (goat anti mouse or anti rabbit HRP, 1∶10,000; Jackson ImmunoResearch, West Grove, PA) for 1 hr at room temperature. Finally, the blot was washed and processed for chemoluminescent detection. We quantified the relative intensities of each signal using the Image J Software and normalized the values to the intensity of Actin.

### Statistical analyses

Statistical analyses were performed using the Sigma Stat program (SigmaStat). The Shapiro-Wilk test was used to determine whether the data were normally distributed, and then statistical significance was calculated using the one sample T-test. Differences with p values less than 0.05 (*p<0.05), less then 0.01 (**p<0.001) and less than 0.001 (***p<0.001) were considered to be significant.

## Supporting Information

Figure S1 Representative digital images of primary fibroblasts from healthy controls and two FRDA patients at basal conditions. Cells nuclei are in Blue (DAPI) and mitochondrial O2- production are in red (MitoSox). Merge is obtained by overlapping the two stain. B. Using digital image processing, the MitoSOX fluorescence intensity mean per image was calculated, averaged over three fields of view per experiment, and then averaged over three independent experiments. Data were normalized to static controls MitoSOX fluorescence. (n = 3, **p<0.01, *p<0.05; Mean +/− SEM) for all the experiments.
(1.05 MB TIF)

Methods S1 Supplementary methods: Fluorescent detection of mitochondrial superoxide.
(0.03 MB DOC)
