# Supplementary figures and images for: PGC-1alpha Down-Regulation Affects the Antioxidant Response in Friedreich's Ataxia
Source: PLoS One. 2010 Apr 7;5(4):e10025. doi: 10.1371/journal.pone.0010025 (PMC2850922; doi:10.1371/journal.pone.0010025)

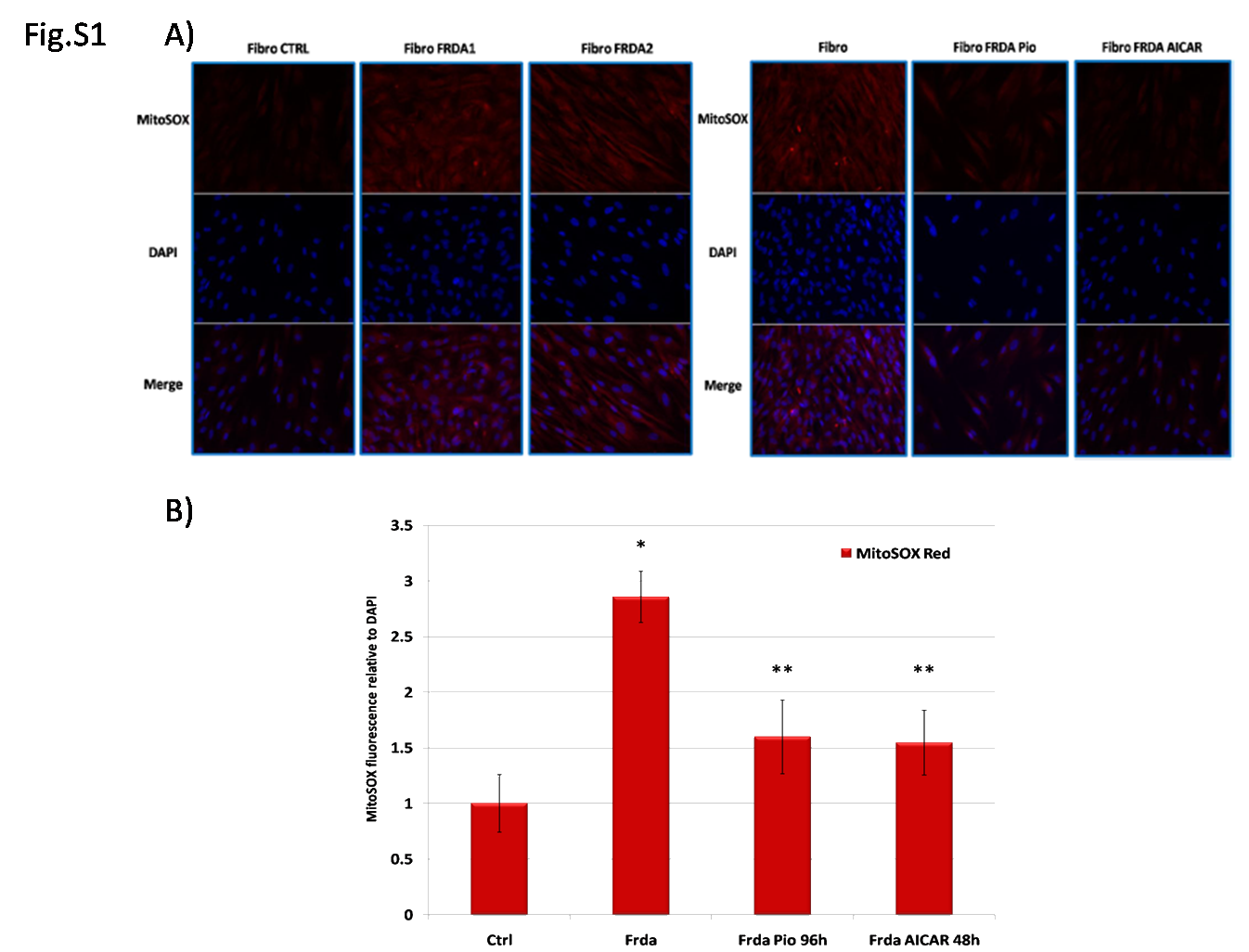

Supplement: Figure S1 — Representative digital images of primary fibroblasts from healthy controls and two FRDA patients at basal conditions. Cells nuclei are in Blue (DAPI) and mitochondrial O2- production are in red (MitoSox). Merge is obtained by overlapping the two stain. B. Using digital image processing, the MitoSOX fluorescence intensity mean per image was calculated, averaged over three fields of view per experiment, and then averaged over three independent experiments. Data were normalized to static controls MitoSOX fluorescence. (n = 3, **p<0.01, *p<0.05; Mean +/− SEM) for all the experiments. (1.05 MB TIF) [file pone.0010025.s001.tif]
