# Supplementary material for: PGC-1alpha Down-Regulation Affects the Antioxidant Response in Friedreich's Ataxia
Source: PLoS One. 2010 Apr 7;5(4):e10025. doi: 10.1371/journal.pone.0010025 (PMC2850922; doi:10.1371/journal.pone.0010025)
Supplement: Methods S1 — Supplementary methods: Fluorescent detection of mitochondrial superoxide. (0.03 MB DOC) [file pone.0010025.s002.doc]

**Methods S1** Fluorescent detection of Mitochondrial O_2_^-^.

MitoSOX Red (514-nm/585-nm, peak excitation and emission, respectively) was used to detect mitochondrial O_2_^-^ and 4’, 6’-diamidino-2-phenylindole (DAPI; 359-nm /461-nm, peak excitation and emission, respectively) to label cell nuclei (Invitrogen, Carlsbad, CA). MitoSOX Red is a mitochondria-targeted form of dihydroethidium that is relatively specific for O_2_^-^and undergoes oxidation to form the DNA-binding red fluorophore ethidium bromide (Tarpey et al., 2004). Cover slips monolayer cultured cells were incubated with MitoSOX red (5μM) for 10 minutes, fixed in formaldehyde 4% and rinsed in PBS before DAPI (1 μM) incubation for 10 minutes. At the end cells were washed and mounted using the FluoroSave^TM^ reagent (Calbiochem)**.** Preparations were observed for fluorescence on a AxioImager™ Z1 fluorescent microscope (Zeiss, Iena, Germany) equipped with an objectives 20x Plan Apochromat 20x/0.8 N.A. Excitation was provided by a HBO 100W mercury lamp. Narrow band-pass filter sets (Zeiss) #49 and #43 were used to visualize the blue and red fluorochromes, respectively. (Details of filter sets: <https://www.micro-shop.zeiss.com/us/us_en/spektral.php?f=fi>). Images (1388 by 1040 pixels) were acquired sequentially for each channel using an AxioCam™ MRm monochrome camera (Zeiss), saved as 12 bit proprietary *.zvi files (Zeiss), processed with AxioVision™ (4.6) software (Zeiss) and exported as uncompressed .jpg files. Figures were prepared with Paint.NET.

Image analysis: images from three cover slips (three images for cover slip) were collected per experiment. After background subtraction, the mean fluorescence intensity per image was calculated and averaged over the three images, using the AxioVision software.
